# Phase-Rotated Altermagnets as Chern Valves for Topological Transport

**DOI:** 10.1021/acs.nanolett.5c05390

**Published:** 2026-02-04

**Authors:** Carlos Caro, Francisco Gámez

**Affiliations:** † Department of Clinical Medicine, Faculty of Health Sciences, 196698 UiT—The Arctic University of Norway, 9037 Tromsø, Norway; ‡ Departamento de Química Física, Facultad Ciencias Químicas, 16734 Universidad Complutense de Madrid, E-28040 Madrid, Spain

**Keywords:** topological transport, Chern channels, angular mass, thermoelectric Hall, nanoelectronic valve

## Abstract

Motivated by the emerging control of Berry-curvature textures in altermagnets, we explore a two-terminal configuration where a topological-insulator film is interfaced with two altermagnetic electrodes whose crystalline phases can be rotated independently. The proximity coupling imprints each altermagnet’s momentum-dependent spin texture onto the Dirac surface states, giving rise to an angular mass whose sign follows the lattice orientation. Adjusting the phase of one electrode redefines this mass pattern, thereby tuning the number and spatial distribution of chiral edge channels. This results in discrete conductance steps and a reversible inversion of the thermoelectric Hall coefficient—achieved without external magnetic fields or net magnetization. A compact Dirac model captures both the quantized switching and its resilience to moderate disorder. Overall, this symmetry-driven mechanism provides a practical and low-dissipation route to programmable topological transport via lattice rotation.

## Graphical abstract

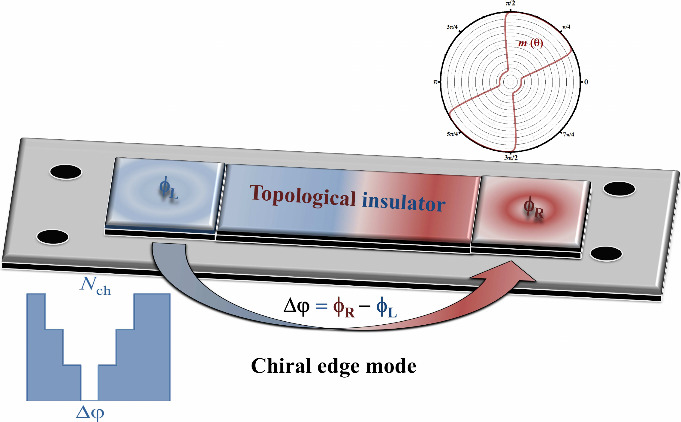

Topological insulators (TIs) represent a central platform for investigating quantum phases that support symmetry-protected edge transport and quantized Hall phenomena.^1,2^ The first observations of the quantum anomalous Hall effect in magnetically doped TIs firmly established the relationship between symmetry breaking and topological conduction, opening the door to broader families of topological matter, and subsequent developments extended this paradigm to higher-order TIs^3,4^ as well as to Floquet-engineered and strain-tunable systems.^5–7^ The ability to manipulate band topology through mechanical or crystalline degrees of freedom now motivates alternative approaches that do not rely on external magnetic fields. A recent and rapidly growing frontier is altermagnetism, a collinear spin order with zero net magnetization but strong momentum-dependent spin splitting enforced by crystalline symmetry.^8,9^ Altermagnetic materials such as RuO_2_, MnTe, and Ca_3_Ru_2_O_7_ display large anomalous Hall effects and strong Berry-curvature multipoles without ferromagnetism.^10,11^ These multipoles, rooted in the *C*_2_ and *C*_4_ symmetry of the lattice, can be reoriented by mechanical strain or shear,^12,13^ offering a route to rotate the underlying spin texture *in situ*. Magnetic-proximity experiments on (Bi_(1–*x*)_Sb*x*)_2_Te_3_ interfaces^15^ demonstrate that symmetry-controlled exchange fields can open directional Dirac gaps of a few millielectronvolts, directly linking spin texture and topological transport. Our proposal is complementary to other recent work on altermagnet-based spintronics. In particular, De la Barrera and Núñez have analyzed electrical control of the exchange bias effect at model ferromagnet–altermagnet junctions, where the altermagnet acts as a collinear antiferromagnetic pinning layer with spin-split bands and the main observable is the hysteretic response of the ferromagnet.^14^ Those proximity geometries focus on tuning the effective exchange field acting on a ferromagnet by modifying the altermagnetic order at the interface. In contrast, in the Chern-valve geometry considered here the altermagnets couple to the surface Dirac states of a three-dimensional TI and are used as symmetry-selective sources of an angular mass. The key control knob is the relative crystalline phase between two independently rotatable altermagnetic contacts, which allows us to switch the integer Chern-channel count at fixed chemical potential and without applying external magnetic fields. In this sense our mechanism provides a dynamically reconfigurable, field-free route to topological transport control that is conceptually distinct from existing altermagnetic exchange-bias and spin-valve proposals. Here we combine these ideas into a minimal and experimentally accessible concept: a two-terminal *Chern valve* in which a TI layer is coupled to two altermagnetic electrodes with independently rotatable crystalline phases ϕ_L_ and ϕ_R_. The operating principle is illustrated in Figure 1a: a TI strip contacted by two altermagnets (AMs) transfers, via spin–orbit proximity, their symmetry-dependent spin textures to the Dirac surface states, generating an angular mass *m*(θ;ϕ) whose sign alternates with the local lattice orientation. A finite phase offset Δφ = ϕ_R_ – ϕ_L_ creates regions where *m*_L_(θ)*m*_R_(θ) < 0, fulfilling the Jackiw–Rebbi criterion^16,17^ and hosting chiral one-dimensional channels at the interfaces.

**Figure 1:**
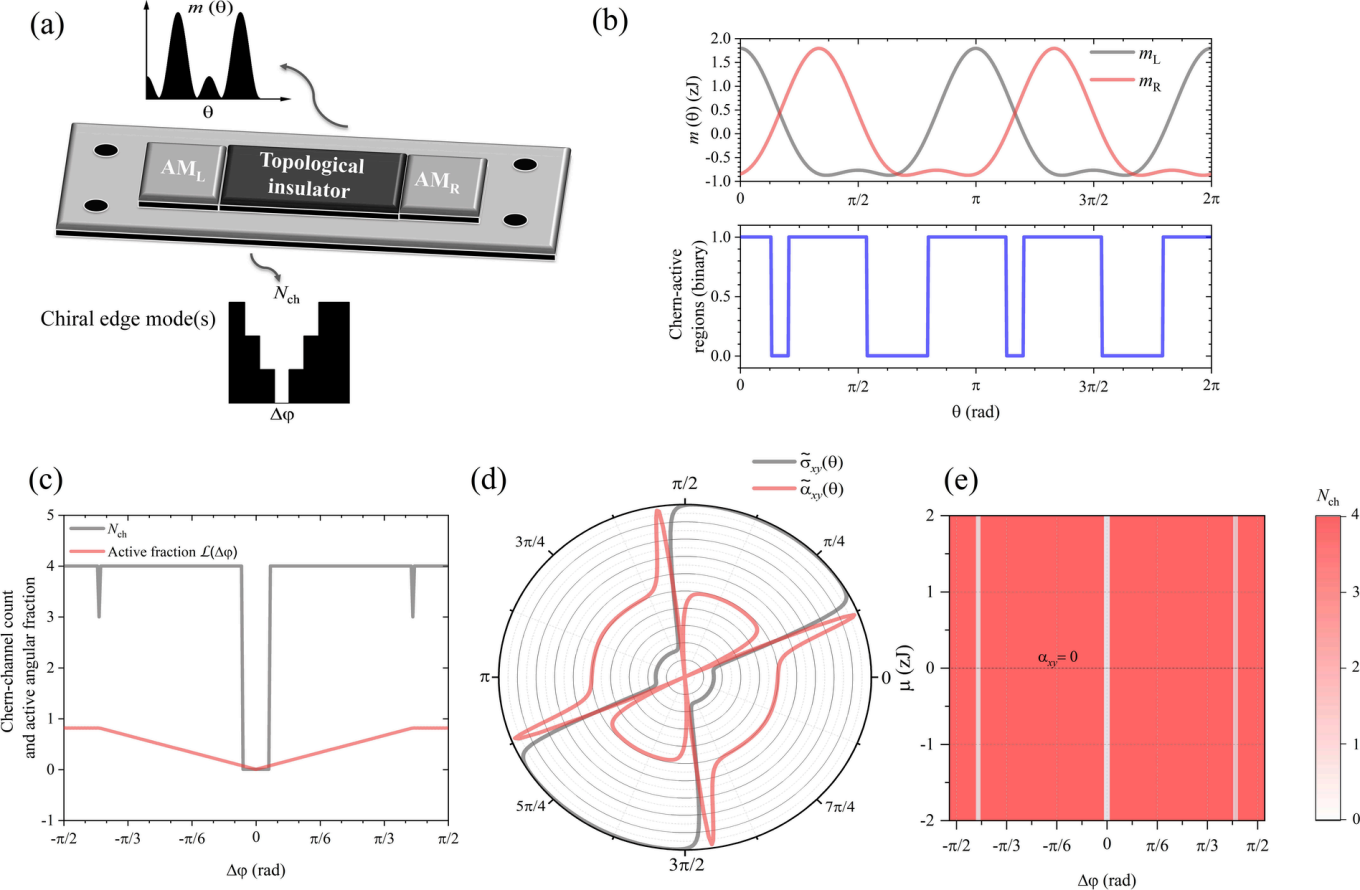
Chern-valve concept and angular-mass topology. (a) Schematic of the AM|TI|AM junction with altermagnets at crystalline phases φ_L_ and φ_R_ inducing angle-dependent masses *m*_L/R_(θ) on the Dirac surface states. A sign inversion across an interface (*m*_L_ *m*_R_ < 0) creates a chiral edge mode (red arrow). (b) Upper panel: angular masses *m*_L_(θ) and *m*_R_(θ) for Δφ = φ_R_ – φ_L_ = π/3, showing two principal lobes reflecting the *C*_2_ and *C*_4_ harmonics. Lower panel: binary signal marking Chern-active sectors where *m*_L_(θ) *m*_R_(θ) < 0. (c) Integer Chern-channel count *N*_ch_ (gray, step-like) and active angular fraction L(Δφ) (red, continuous) as a function of phase difference Δφ, showing quantized plateaus. The fraction L is normalized such that L=1 corresponds to full 2π angular coverage. Numerical simulations employ representative parameters for RuO_2_—Bi_2_Se_3_ interfaces: *m*_2_ ≈ 2 meV, *m*_4_ ≈ 0.5 meV, Fermi velocity *v*_F_ = 5 × 10^5^ m/s, and temperature *T* = 15 K. (d) Normalized anomalous Hall and thermoelectric Hall conductivities σ̃_*xy*_(φ) and α̃_*xy*_(φ) exhibiting *C*_2_/*C*_4_ harmonic phase shifts locked to φ, with σ̃_*xy*_(φ) = σ_*xy*_(φ)/max_φ_|σ_*xy*_| and α̃_*xy*_(φ) = α_*xy*_(φ)/max_φ_|α_*xy*_|. (e) Phase diagram of the Chern-valve response *N*_ch_(μ,Δφ) across the (μ, Δφ) plane. White regions correspond to *N*_ch_ = 0 (aligned altermagnetic masses without sign inversion), while red regions denote finite *N*_ch_, where chiral edge channels are active; the color scale encodes the integer channel count. The purple contour marks the condition α_*xy*_^tot^ = 0, separating regions of opposite transverse thermoelectric polarity. This map demonstrates that the quantized channel topology and the thermo-Hall polarity remain robust over a wide range of electrochemical conditions.

The essential physics is captured by a minimal Dirac model describing a two-dimensional TI surface proximized by two altermagnets with independently rotated crystalline phases:

$$H_{\mathrm{TI}}\left(k\right)=\hbar v_{F}\left(k_{x}\sigma_{y}-k_{y}\sigma_{x}\right)+m\left(\theta ;\phi \right)\;\sigma_{z}$$ 1

where **k** = (*k*_*x*_, *k*_*y*_) is the crystal momentum, θ = a tan 2­(*k*_*y*_, *k*_*x*_) the azimuthal angle, *v*_F_ the Fermi velocity, and σ_*x*,*y*,*z*_ are Pauli matrixes. Because typical altermagnets exhibit *C*_2_ or *C*_4_ spin-rotation symmetries, their Berry-curvature multipoles map onto the harmonic angular dependence of the mass term:

$$m\left(\theta ;\phi \right)=m_{0}+m_{2}\;\cos\left[2\left(\theta -\phi \right)\right]+m_{4}\;\cos\left[4\left(\theta -\phi \right)\right]$$ 2

where *m*_0_, *m*_2_, and *m*_4_ are real amplitudes proportional to the interfacial exchange and spin–orbit coupling strength. The *m*_2_ and *m*_4_ coefficients encode the dipolar (*C*_2_) and quadrupolar (*C*_4_) Berry-curvature multipoles of the altermagnet. In the **d**-vector notation with **d**(**k**;ϕ) = [−*ℏv*_F_ *k*_*y*_,*ℏv*_F_ *k*_*x*_,*m*(θ;ϕ)], the band-resolved Berry curvature is

$$\Omega_{\;\pm ,z}\left(k;\theta \right)=\mp \frac{1}{2}\;\frac{d\cdot \left(\partial_{k_{x}}d\times \partial_{k_{y}}d\right)}{|d{|}^{3}}$$ 3

The intrinsic anomalous Hall conductivity follows from integrating the Berry curvature over the Brillouin zone:

$$\sigma_{xy}=-\frac{e^{2}}{\hbar }\underset{n=\pm }{\sum }\int_{\mathrm{BZ}}\frac{d^{2}k}{{\left(2\pi \right)}^{2}}f\left(\varepsilon_{nk},\mu ,T\right)\;\Omega_{n,z}\left(k\right)$$ 4

where ε_*n* **k**_ is the energy dispersion and *f*(ε_*n* **k**_,μ,*T*) is the Fermi–Dirac distribution. The thermoelectric Hall coefficient satisfies the low-temperature Mott relationship:

$$\alpha_{xy}=-\frac{\pi^{2}{k_{B}}^{2}T}{3e}{\left(\frac{\partial \sigma_{xy}}{\partial \mu }\right)}_{T\to 0}$$ 5

which holds for *T* ≲ 10 K in typical AM—TI stacks. Because *m*(θ;ϕ) rotates rigidly with the crystalline phase, both σ_*xy*_ and α_*xy*_ inherit its *C*_2_/*C*_4_ symmetry, providing a direct symmetry-protected electrical and thermoelectric readout of the Chern-active regions.

The quantized topological channels emerge when the angular mass changes sign between the two altermagnetic contacts. Figure 1(b) illustrates how the relative crystalline phase Δφ controls chiral-sector formation in the TI channel. The upper panel shows the angular masses *m*_L_(θ) and *m*_R_(θ), each displaying two principal positive and negative lobes within a 2π cycle, reflecting the superposition of *C*_2_ and *C*_4_ harmonics. With the red curve (*m*_R_) offset by Δφ = π/3 from the gray one (*m*_L_), the zeros alternate along θ, creating four regions where *m*_L_(θ) and *m*_R_(θ) have opposite signs. The lower panel marks the Chern-active sectors where *m*_L_(θ) *m*_R_(θ) < 0. For each θ, this binary signal identifies sectors satisfying the Jackiw–Rebbi condition and hosting one-dimensional chiral edge states.

The number of active sectors is obtained by counting the sign reversals:

$$N_{\mathrm{ch}}=\mathrm{nint}\left[\frac{1}{2\pi }\int_{0}^{2\pi }d\theta \;\Theta \left[-m_{L}\left(\theta \right)\;m_{R}\left(\theta \right)\right]\right]$$ 6

with the continuous active angular fraction:

$$L\left(\Delta \varphi \right)=\frac{1}{2\pi }\int_{0}^{2\pi }d\theta \;\Theta \left[-m_{L}\left(\theta \right)\;m_{R}\left(\theta \right)\right]$$ 7

where Θ­(*x*) denotes the Heaviside step function, with Θ­(*x*>0) = 1 and Θ­(*x*≤0) = 0, and that nint­[*x*] returns the nearest integer to *x*. In practice, we evaluate *N*_ch_ by discretizing θ into *N*_θ_ points and counting the connected angular sectors where *m*_L_(θ) *m*_R_(θ) < 0 with a minimal width *w*_min_, which is equivalent to eq 6 in the continuum limit. For Δφ = π/3, four “on” intervals appear, giving *N*_ch_ = 4 and L≈0.5 , meaning roughly half the Fermi contour contributes to topological transport.

Figure 1c shows the phase-offset dependence. The gray trace (integer *N*_ch_) remains at four for most offsets, collapsing to zero near Δφ = 0 where the masses are aligned and no sign inversion occurs. Small notches where *N*_ch_ = 3 appear near Δφ = ±π/3, when two sign-changing boundaries merge into a tangential zero of *m*_L_(θ) *m*_R_(θ), momentarily suppressing one active sector. The red curve ( L ) varies smoothly, reaching its minimum at alignment. Both observables reveal the same mechanism: as the relative crystalline phase increases, additional opposite-sign sectors emerge sequentially, enabling stepwise tuning of the topological channels.

Figure 1d displays the angular dependence of the intrinsic Hall responses. Both the anomalous Hall and thermoelectric Hall coefficients (normalized to emphasize relative phase and symmetry) follow the 4-fold *C*_2_/*C*_4_ pattern as the mass term but shift in phase with ϕ, providing a direct symmetry-locked electrical and thermoelectric signature and confirming their common Berry-curvature origin through the coincidence of peaks with angular sectors where *m*_L_ *m*_R_ < 0.

The robustness of the topological quantization across the full accessible range of chemical potentials and phase offsets is captured in Figure 1e, which displays a phase diagram mapping *N*_ch_(μ,Δφ) across the (μ, Δφ) plane. The diagram reveals quantized plateaus as a function of both the Fermi level (chemical potential μ) and the relative crystalline phase. The white regions correspond to *N*_ch_ = 0, where the left and right altermagnetic masses are aligned and no sign inversion occurs, whereas the red sectors indicate finite *N*_ch_ representing active chiral-valve configurations. The purple contour marks the condition α_*xy*_^tot^ = 0, separating regions of opposite transverse thermoelectric polarity. This map demonstrates that the quantized channel topology and its associated thermo-Hall response remain robust over a wide range of electrochemical conditions, which is crucial for device operation.

The proposed geometry can be implemented using established thin-film growth and strain-control techniques. High-quality topological-insulator Bi_2_Se_3_ films (50–100 nm) can be grown by molecular beam epitaxy on various substrates, with excellent structural quality demonstrated through rocking-curve line widths below 15 arcsec and clear layer thickness fringes.^21,22^ For the altermagnetic contact layers, RuO_2_ (10–20 nm) and α-Fe_2_O_3_ (hematite) films are deposited by pulsed-laser deposition on compatible oxide substrates. The lattice matching between Bi_2_Se_3_ and typical substrates (SrTiO_3_, InP, Al_2_O_3_) exhibits lattice mismatch below 3%, supporting coherent heteroepitaxial growth via strain-mediated van der Waals interactions.^23,24^ Independent in-plane strain control can be achieved through piezoelectric or flexible substrates and actuators. Experimental observations confirm that controlled strain of ≈1% is sufficient to induce phase-dependent changes in collinear magnetic order in both RuO_2_ and MnTe, with such strain amplitudes being reversible and nonhysteretic across multiple cycles.^18–20^ This level of strain is achievable through standard strain-engineering techniques in oxide heterostructures and constitutes an experimentally realistic switching mechanism for the proposed Chern valve. To quantify the required crystalline rotation and strain, we have evaluated the channel count *N*_ch_(Δφ) as a function of the relative phase between the two altermagnets, Δφ = φ_R_ – φ_L_, using the same representative parameters as in Figure 1. Imposing a minimal angular width of *w*_min_ = 6° for a conducting sector, we find that the Chern valve remains in a fully blocked regime (*N*_ch_ = 0) for |Δφ| ≲ 6°, while a four-channel state with *N*_ch_ = 4 emerges for |Δφ| ≳ 6.5°. In other words, a relative misalignment of the Néel vectors by only ∼5–10° is sufficient to switch between the “off” and “on” topological plateaus. Recent experimental and theoretical works on epitaxial RuO_2_ and MnTe indicate that uniaxial strains of order ε ∼ 1% can already drive a repopulation of domains and a rotation of the altermagnetic spin texture by angles of the order of a few tens of degrees.^18–20^ Within this range, a full phase difference Δφ ≃ π/2, sufficient to traverse one conductance plateau, would correspond to strains of order ε ∼ 2–3%, still well within the elastic window of oxide and chalcogenide thin films on piezoelectric substrates. Additionally, using DFT-based Wannier tight-binding parameters for RuO_2_ ^10^ and experimental estimates of proximity-induced exchange gaps in magnetically gapped Bi_2_Se_3_ surface states,^15^ we can obtain a simple order-of-magnitude estimate for the angular mass in a RuO_2_—Bi_2_Se_3_ heterostructure. Taking a representative interfacial exchange splitting Δ_ex_ ≈ 5–10 meV, a **k**·**p** downfolding of the bulk altermagnetic spin texture onto the TI surface yields harmonic amplitudes of order *m*_2_ ≃ 0.6Δ_ex_ and *m*_4_ ≃ 0.3Δ_ex_. For the above range of Δ_ex_, this gives *m*_2_ ≈ 3–6 meV and *m*_4_ ≈ 1.5–3 meV, i.e., millielectronvolt-scale masses fully consistent with the parameters used in our simulations and compatible with the Dirac-gap sizes reported in refs 15, 21, and 22. This confirms that the angular masses required for Chern-valve operation are achievable, for instance, in realistic RuO_2_—Bi_2_Se_3_ devices. We emphasize that the strain amplitudes considered here, ε ∼ 1–2%, correspond to in-plane epitaxial or piezoelectric strain used to reorient the altermagnetic crystalline phase, rather than to drive a bulk topological transition in the TI itself. In our description the three-dimensional TI remains in the same *Z*_2_ topological phase throughout; strain only enters via the altermagnets, by modifying the orientation and magnitude of the proximity-induced angular mass *m*(θ;φ) at the surface. The bulk Dirac gap and Fermi velocity of the TI are kept fixed and well within the topological regime, so that the Chern-valve operation is entirely controlled by boundary conditions. Much stronger or nonuniform deformations, such as large out-of-plane strain capable of closing and reopening the bulk gap of the TI, could in principle trigger a separate topological phase transition, but such regimes lie outside the operating window of the present proposal.

In the phase-rotated AM|TI|AM junction, transport within the interfacial gap is carried by one-dimensional chiral channels that appear whenever the angular masses have opposite sign, *m*_L_(θ) *m*_R_(θ) < 0. For fixed (μ, Δφ), let *N*_ch_(μ,Δφ) be the number of such channels. The two-terminal conductance in the Landauer picture reads

$$G\left(\mu ,\Delta \varphi ,T\right)=\frac{e^{2}}{h}\overset{N_{\mathrm{ch}}\left(\mu ,\Delta \varphi \right)}{\underset{i=1}{\sum }}T_{i}\left(\mu ,T\right)$$ 8

where $T_{i}$ is the transmission of channel *i*. In the clean, low-temperature limit and for well-matched contacts we have $T_{i}\to 1$ ; hence,

$$G\left(\mu ,\Delta \varphi ,0\right)=\frac{e^{2}}{h}N_{\mathrm{ch}}\left(\mu ,\Delta \varphi \right)$$ 9

As the relative crystalline phase Δφ is tuned, the set of angles θ that satisfy *m*_L_(θ) *m*_R_(θ) < 0 changes discretely: each creation/annihilation of a Chern-active sector adds/removes one chiral mode, producing conductance steps of height Δ*G* = *e* ^2^/*h*. No additional factor of 2 appears because each chiral channel is singly degenerate (the exchange-induced gap on the TI surface breaks Kramers degeneracy and lifts spin doubling). At finite temperature or with moderate disorder, $T_{i}<1$ and the plateaus acquire a slight slope, but their spacing in units of *e* ^2^/*h* remains set by *N*_ch_. Consequently, two-terminal conductance measurements should reveal discrete plateaus separated by *e* ^2^/*h* as the relative crystalline phase is tuned through strain, in accordance with eqs 8 and 9. Simultaneous thermoelectric characterization using standard microheater and thermometer geometries can detect the predicted sign inversion of α_*xy*_. The absence of net magnetization eliminates parasitic Hall offsets and stray-field effects, allowing the geometric nature of the switching to be isolated unambiguously. The channel-switching energy scale of order 1 meV implies operational temperatures up to ∼15 K (consistent with *k*_B_ *T* ≲ 1 meV), accessible with standard cryogenic setups. Reversible piezoelectric actuation enables dynamic tuning rates in the microsecond range, making the device suitable for low-power, symmetry-controlled topological logic elements. Although we have illustrated the Chern-valve mechanism using *C*_2_/*C*_4_ altermagnets, the construction is not restricted to these symmetries. For a *C*_6_ altermagnet the angular mass would acquire an additional harmonic of the form *m*_6_ cos­[6­(θ – φ)], leading to six positive and six negative lobes of *m*(θ;φ) around the Fermi contour. A finite phase offset Δφ between two such contacts still produces angular sectors where *m*_L_(θ)*m*_R_(θ) < 0 and thus hosts chiral interface channels; only the number and angular width of the Chern-active sectors change compared to the *C*_2_/*C*_4_ case. A quantitative analysis of *N*_ch_(Δφ) for concrete *C*_6_ altermagnets is left for future work, but the symmetry considerations that underlie the Chern-valve mechanism apply equally to recently identified hexagonal altermagnets. Several practical limitations merit explicit discussion. First, our minimal Dirac model assumes sharp interfaces and an angular mass containing only the leading *C*_2_/*C*_4_ harmonics. In this description the robustness of the Chern-valve plateaus is controlled primarily by the sign structure of *m*(θ) and by the presence of an interfacial gap, rather than by a fine-tuning of individual harmonic amplitudes. In a real device, symmetry-breaking distortions, including higher-order harmonics (*m*_6_, *m*_8_,...), interface roughness, interdiffusion and intermixing at the AM|TI boundaries will inevitably introduce scattering and distort the Berry-curvature texture. Moderate distortions of this kind deform the angular regions where *m*_L_(θ)*m*_R_(θ) < 0, broadening the switching transitions in the phase diagram of Figure 1e and reducing the transmission of individual channels ( $0<T_{i}<1$ ), so that the plateaus become slightly rounded while their spacing in units of *e* ^2^/*h* remains fixed by the integer channel count *N*_ch_. A fully microscopic treatment of strong disorder in the AM and TI regions, including explicit band-structure or scattering-matrix calculations, lies beyond the present scope but would be highly valuable for device-level optimization.

Second, the predicted switching energy scale (≈ 1 meV) sets the operational temperature limit to *T* ≲ 15 K, constraining practical device deployment to cryogenic platforms. This limitation is shared with other geometric topological switches and is still less stringent than for many superconducting approaches. Third, achieving independent strain control across both altermagnetic contacts demands either separate piezoactuators (increasing complexity and power) or spatially resolved strain patterning via lithography, both of which are technologically feasible but will require further optimization. Fourth, the model neglects lattice imperfections, point defects and thermal magnon excitations in the altermagnet, which can renormalize the exchange coupling and partly mask the predicted quantisation under ambient conditions. Fifth, the Fermi-level tunability shown in Figure 1e assumes clean charge-carrier accumulation; in real devices, band-bending, trap states and back-gate leakage will distort the μ­(Δφ) map and reduce the visibility of individual plateaus. Despite these challenges, the underlying protection mechanism, rooted in Jackiw–Rebbi zero-mode formation and in the topological sign structure of the mass, is robust to small perturbations that preserve the primary *C*_2_ or *C*_4_ symmetry and keep the interfacial gap open. Experimental demonstration will be crucial to determine the actual quantisation tolerance margins and to refine material choices and growth protocols.

The Chern valve represents a distinct switching paradigm compared to established mechanisms. Magnetic-field-controlled Chern insulators demand strong external fields and typically exhibit hysteretic behavior,^1^ while Floquet topological engineering requires high-frequency optical modulation with inherent dissipation.^5,6^ This approach achieves fully reversible control with minimal dissipation via static lattice rotation. Table 1 compares operational characteristics and literature benchmarks.^1,5,6,25,26^ Extensions of this concept stablish a route toward strain-tunable topological logic and spin–orbit device platforms.

**Table 1:** Comparison of Topological-Switching Mechanisms and Their Experimental Benchmarks

feature	magnetic field^1^	Floquet drive^5,6^	this work (strain/rotation)
dissipation	high (Oersted, eddy)	moderate–high (optical)	minimal (geometric)
speed	ms range	fs–ps range	μs range (piezo)
reversibility	often hysteretic	periodic	fully reversible
operation temperature	1–300 K	variable	<15 K
magnetization	yes	no	no
physical handle	magnetic field	light	lattice rotation (strain)

## Numerical Grid and Convergence Details

All numerical simulations were performed on dense, symmetry-adapted grids to ensure full convergence of both the topological and thermoelectric quantities. The momentum-space integrals were computed using *N*_*k*_ = 300, *N*_θ_ = 601, and *N*_Δϕ_ = 241, uniformly sampling the Brillouin zone and the interlayer phase difference. The chemical potential was discretized into *N*_μ_ = 151 covering either the gapped window [−6, 6] meV or the extended range [−15, 15] meV used in the wide-band phase diagrams. The angular integration over the scattering plane was limited to a minimal width of *w*_min_ = 6°, which ensures stability of the polar plots. Convergence tests were carried out by doubling the sampling densities (*N*_*k*_, *N*_θ_), leading to variations below 10^–3^ in all integrated quantities. All figures presented in this work correspond to these numerical parameters unless explicitly stated otherwise.
